# Curative resection via right hemicolectomy and regional lymph node dissection for colonic adenomatous polyposis of unknown etiology with adenocarcinomas localized in the right side of the colon: a case report

**DOI:** 10.1186/s40792-024-01890-1

**Published:** 2024-04-22

**Authors:** Shu Aoyama, Akira Inoue, Yoshinori Kagawa, Takamichi Komori, Yuki Ozato, Yujiro Nishizawa, Tomoki Sugimoto, Hisateru Komatsu, Masashi Hirota, Yasuhiro Miyazaki, Akira Tomokuni, Masaaki Motoori, Hiroaki Fushimi, Gou Yamamoto, Kiwamu Akagi, Kazuhiro Iwase, Kazumasa Fujitani

**Affiliations:** 1 Department of Gastroenterological Surgery, Osaka General Medical Center, 3-1-56 Bandaihigashi, Sumiyoshi-Ku, Osaka, Japan; 2 Department of Surgery, Hyogo Prefectural Nishinomiya Hospital, Nishinomiya, Japan; 3 Department of Pathology, Osaka General Medical Center, Osaka, Japan; 4 Division of Molecular Diagnosis and Cancer Prevention, Saitama Cancer Center, Kitaadachi-gun, Japan

**Keywords:** Colonic adenomatous polyposis of unknown etiology, Colorectal cancer, *MSH3*

## Abstract

**Background:**

*APC* and *MUTYH* are both well-known colorectal polyposis causative genes. However, 30–50% of colorectal adenomatous polyposis cases are classified as colonic adenomatous polyposis of unknown etiology and lack identifiable pathogenic variants. Although guidelines recommend total proctocolectomy for colonic adenomatous polyposis of unknown etiology with over 100 adenomas, evidence is lacking. This study presents a unique case of localized colonic adenomatous polyposis of unknown etiology with multiple adenocarcinomas, treated with hemicolectomy and regional lymph node dissection.

**Case presentation:**

The patient was a 72-year-old woman whose colonoscopy revealed numerous polyps and two adenocarcinomas localized in the right side of the colon, with no lesions in the left side. The patient had no family history of polyposis or colorectal cancer. No extracolonic lesions, enlarged lymph nodes, or distant metastases were found. Considering the patient’s age and lesion localization, laparoscopic right hemicolectomy with regional lymph node dissection was performed. Histopathological diagnosis revealed three adenocarcinoma lesions with no lymph node metastasis. The most advanced pathological stage was T2N0M0 Stage I (UICC 8th edition). The patient was alive 5 years postoperatively, without recurrence of cancer or polyposis in the remaining colon and rectum. To diagnose hereditary colorectal cancer/polyposis, a germline multigene panel testing for *APC, EPCAM, MBD4, MLH1, MLH3, MSH2, MSH3, MSH6, MUTYH, NTHL1, PMS2, POLD1, POLE,* and *TP53* was performed using DNA extracted from blood samples: however, no pathogenic variant was detected. Therefore, the patient was diagnosed with colonic adenomatous polyposis of unknown etiology.

**Conclusions:**

In this rare case, colonic adenomatous polyposis of unknown etiology, with numerous adenomatous polyps and multiple adenocarcinomas localized in the right side of the colon, was successfully treated with right hemicolectomy and regional lymph node dissection. Despite genetic analysis, no causative germline variants were identified. Segmental colectomy according to the distribution of polyps might be a curative approach.

## Background

The most common cause of colorectal adenomatous polyposis is hereditary adenomatous polyposis, typically resulting from germline pathogenic variants in the *APC* or *MUTYH* genes. However, the causative germline variants cannot be identified in many cases of colorectal adenomatous polyposis, accounting for as many as 30–50% of polyposis cases [1, 2]. In such cases, the condition is classified as colonic adenomatous polyposis of unknown etiology (CPUE). The National Comprehensive Cancer Network guidelines recommend that the management of CPUE cases wherein cumulative lifetime adenomas exceed 100 should be equivalent to that of familial adenomatous polyposis (FAP), with total proctocolectomy considered the standard treatment [3]. However, no evidence is available to date to support this treatment approach, and the inclusion of various genetic diseases within the CPUE category makes it difficult to establish a uniform treatment strategy [4].

In this report, we present a case of CPUE with numerous adenomas and multiple adenocarcinoma lesions confined to the right side of the colon, wherein right hemicolectomy was performed, and long-term survival was achieved.

## Case presentation

A 72-year-old woman was referred to our hospital owing to numerous polyps in the right side of the colon detected on colonoscopy performed for the examination of fecal occult blood. The patient had several comorbidities, including type 2 diabetes, hypertension, and dyslipidemia, with no history of smoking or alcohol consumption or of treatment for childhood or adult cancers. The patient’s father and brother had lung and gastric cancers, respectively; however, the patient had no family history of colorectal polyposis or cancer.

**Fig. 1:**
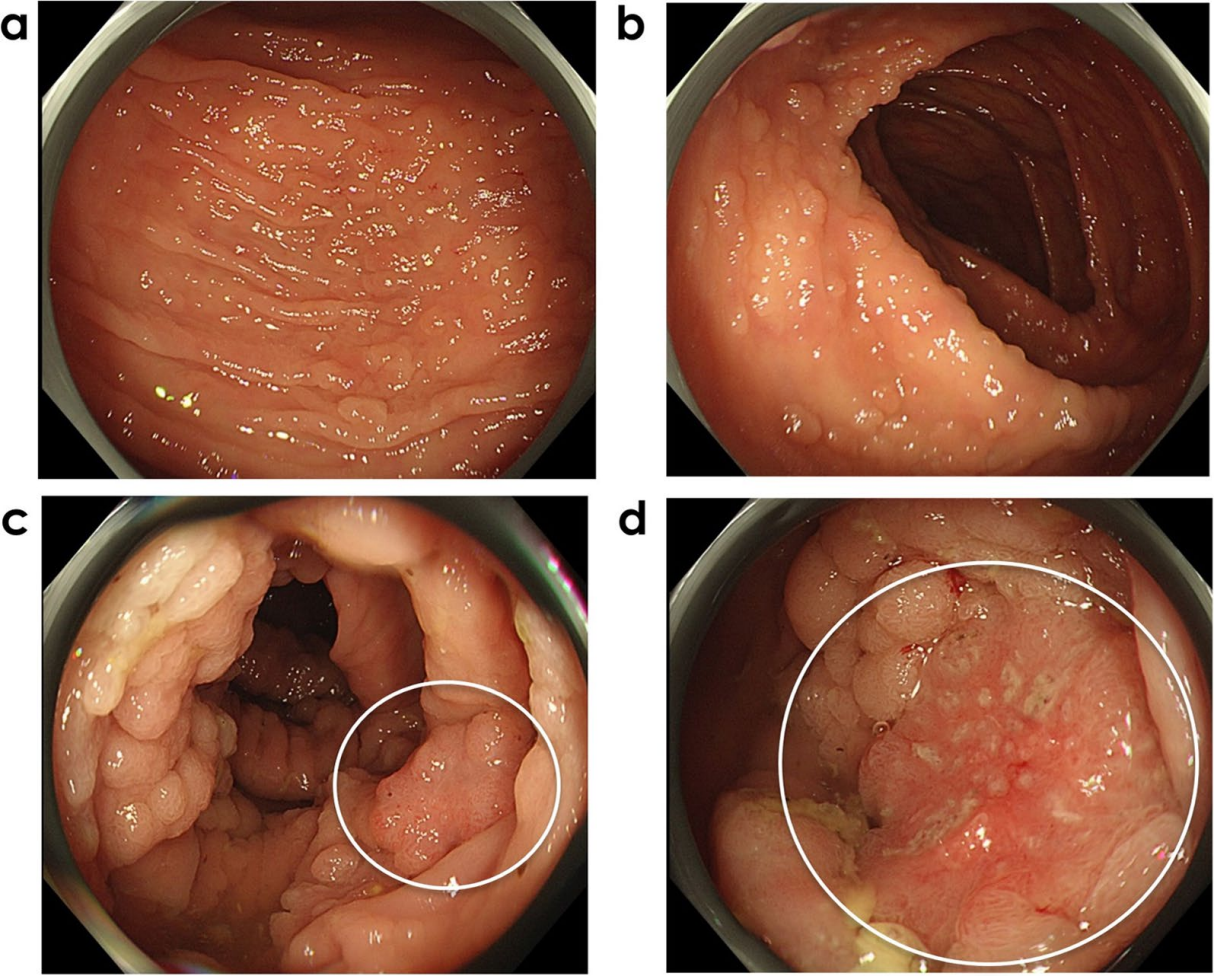
Colonoscopy images of the colon. Numerous adenomatous polyps with a normal mucosal background are observed in **a** the ascending colon and **b** transverse colon. Adenocarcinomas are detected in two lesions surrounded by white circles in the **c** ascending colon and **d** transverse colon

Colonoscopy revealed > 100 polyps, with a normal mucosal background confined from the cecum to the splenic flexure of the transverse colon (Fig. 1a, b). A clinical diagnosis of sparse FAP was made based on guidelines [5, 6]. No polyp lesions were observed in the descending colon, sigmoid colon, or rectum. Biopsy results confirmed that the polyps were adenomas, with two moderately differentiated tubular adenocarcinomas detected in the ascending and transverse colon. The lesion in the transverse colon was suspected of submucosal invasion (Fig. 1c, d). Computed tomography revealed no evidence of enlarged lymph nodes, distant metastases, or other organ lesions. Upper gastrointestinal endoscopy and ocular fundoscopy revealed no evidence of extracolonic lesions.

**Fig. 2:**
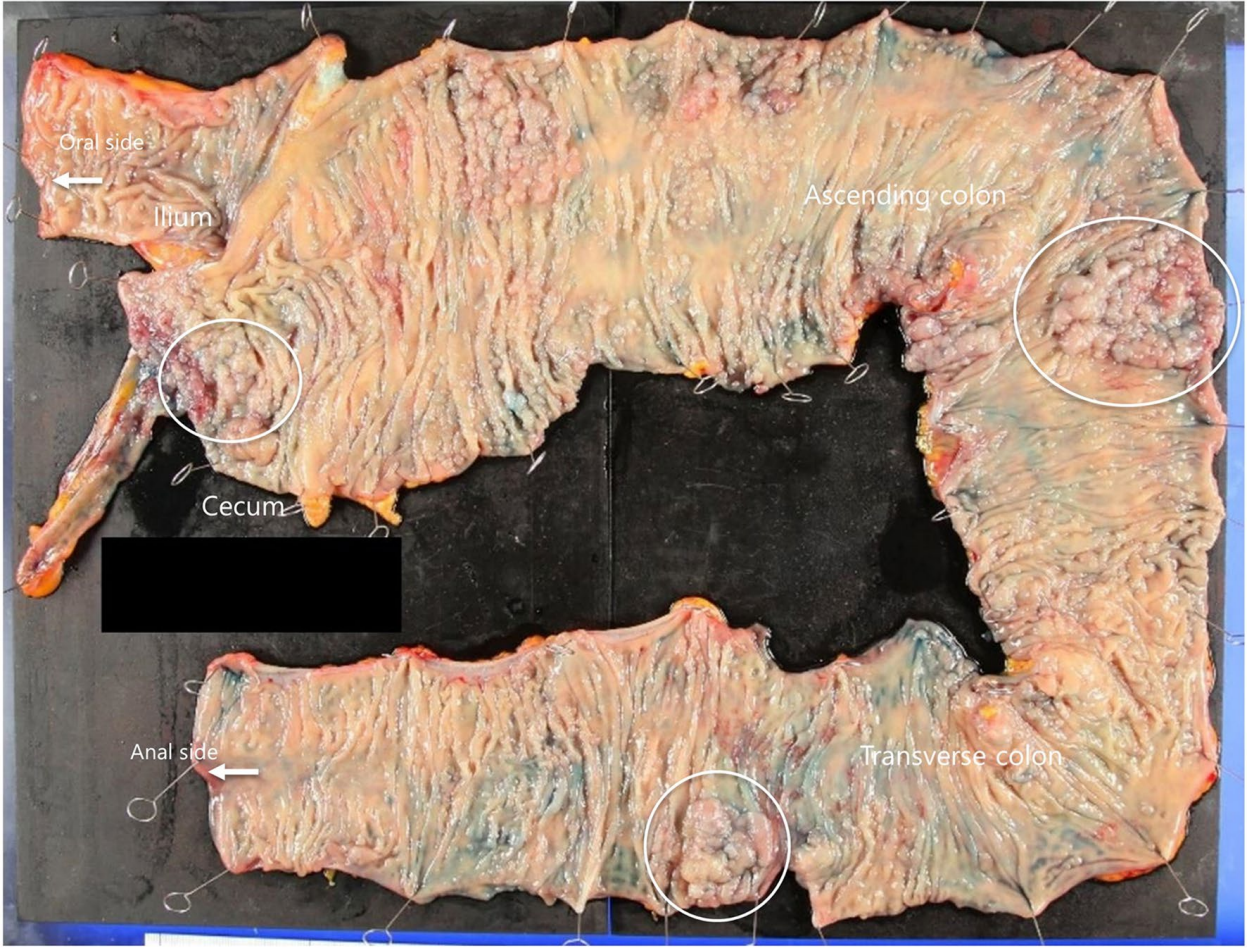
Resected specimen of the right hemicolectomy. Adenocarcinomas are detected in three lesions indicated by white circles

Laparoscopic right hemicolectomy with regional lymph node dissection was performed (Fig. 2), and the patient was discharged on postoperative day 7 without any postoperative complications. Histopathological examination revealed a moderately differentiated adenocarcinoma in the transverse colon with invasion of the muscularis propria and two lesions of highly differentiated intraepithelial adenocarcinoma in the cecum and ascending colon. No lymphatic or venous invasion or lymph node metastasis was observed. Approximately 800 adenomas were diffusely distributed from the ascending colon to the splenic flexure of the transverse colon against a background of normal mucosa, some densely fused, with adenocarcinomas detected in such areas. The pathological stage of the most advanced one was T2N0M0 Stage I according to the eighth edition of the Tumor Node Metastasis Classification of the International Union Against Cancer [7]. Most polyps were tubular adenomas; however, some showed serrated changes. The resection margins showed no neoplastic or adenomatous atypia. The tumors were microsatellite stable. At 5 years postoperatively, the patient remains cancer-free, with no recurrence of cancer or polyposis in the remaining colon or rectum, although few sporadic adenomatous polyps developed in the sigmoid colon.

**Fig. 3:**
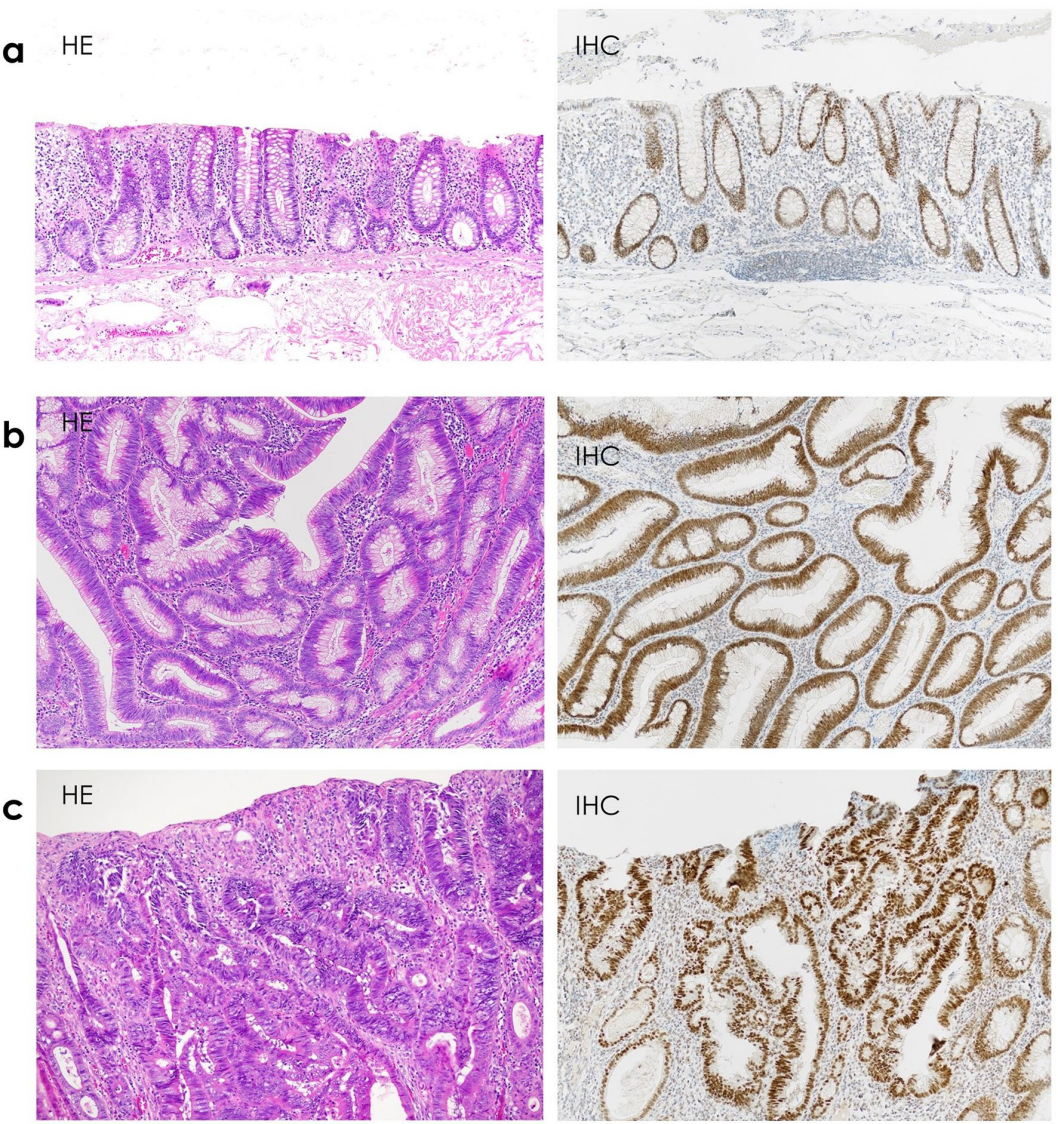
Hematoxylin–eosin staining and immunohistochemistry of MSH3 (all magnifications are 100 ×). The expression of MSH3 is similar among sites. **a** Normal mucosa, **b** adenoma, **c** adenocarcinoma. *HE*, Hematoxylin–eosin staining; *IHC*, immunohistochemistry

To identify causal genetic factors, DNA sequencing of blood samples was performed using an original multigene panel for *APC, EPCAM, MBD4, MLH1, MLH3, MSH2, MSH3, MSH6, MUTYH, NTHL1, PMS2, POLD1, POLE,* and *TP53*. The exons and 5-bp flanking regions of these genes were sequenced and evaluated. For *APC* and mismatch repair genes (*MLH1, MSH2, MSH6,* and *PMS2*), splicing abnormalities were also analyzed in mRNA. The results revealed a heterozygous variant in *MSH3* (c.606G > A, p.Cln202 =), which was interpreted as likely benign in ClinVar [8], and no difference in MSH3 expression was observed between the normal mucosa, adenomas, and carcinomas using tissue immunostaining (Fig. 3) of formalin-fixed, paraffin-embedded specimens with a rabbit monoclonal anti-MSH3 antibody (ab111107; Abcam, Cambridge, UK), performed as described previously [9]. Because serrated lesions were observed in some polyps on hematoxylin and eosin staining, the possibility of serrated polyposis was considered. RNA sequencing of *RNF43* using the blood samples was also performed; however, no variant was found. Based on the above findings, the final diagnosis was CPUE.

## Discussion

Herein, we report a rare case of CPUE with localized lesions, including multiple carcinomas, in the right side of the colon. In contrast to the recommended total proctocolectomy for CPUE cases with over 100 adenomatous polyps, we opted for a right hemicolectomy with regional lymph node dissection, resulting in long-term survival. Despite a comprehensive search for germline variants in genes associated with polyposis diseases, including *APC* and *MUTYH*, no causative germline variant was identified.

While a definitive consensus on the trend in the lesion distribution in CPUE remains elusive, several reports have suggested that lesions are more frequently found on the right side in CPUE cases with a small number of lesions [10]. However, CPUE cases with > 100 lesions distributed in a local area of the colon or rectum have not been reported, other than the present case. With respect to FAP cases with *APC* pathogenic variants, a search of the PubMed database revealed two prior reports of typical FAP cases involving localized lesions. In one case, a complication of ulcerative colitis was reported, and the right side of the colon was covered by innumerable small adenomatous polyps; conversely, ulcerative colitis was observed on the left side of the colon [11]. This patient underwent total abdominal colectomy as a curative treatment for both FAP and ulcerative colitis. In the other case, numerous right-sided polyps were detected, sparing the left side of the colon [12]. In this case, laparoscopic subtotal colectomy was performed, while laparoscopic pancreatoduodenectomy was conducted simultaneously for ampullary cancer. In our case, the patient had > 100 colon adenomas and was diagnosed with CPUE after germline multigene panel testing. Although the condition in our case may have been caused by a variant in a gene that was not included in our multigene panel, to the best of our knowledge, this is the first reported case of localized polyposis lesions in CPUE with > 100 lesions.

According to guidelines [3], the management of CPUE cases with > 100 adenomatous polyps should follow that of FAP; as such, the standard treatment is total proctocolectomy. However, no strong evidence is available to support this treatment approach. Total proctocolectomy is associated with a decreased quality of life [13]. Given that our patient was older, the detrimental impact on quality of life was expected to be even greater. Accounting for the localization of the polyps and adenocarcinomas, we opted to perform segmental resection and regional lymph node dissection of the diseased colon, assuming strict endoscopic surveillance after the surgery. In attenuated FAP cases, endoscopic polypectomy followed by endoscopic surveillance is considered a standard treatment [14]. Therefore, we deemed that our strategy, with strict endoscopic surveillance, would be a feasible approach in our case. Postoperatively, periodic surveillance with colonoscopy for > 5 years revealed no evidence of polyposis or cancer recurrence in the remaining colon or rectum. Even in cases of CPUE with > 100 lesions, curative resection might be achieved by resecting the diseased bowel (with lymph node dissection in cases with adenocarcinomas) instead of performing total proctocolectomy when the lesion is localized, as in the present case.

In addition to FAP and *MUTYH*-associated polyposis, colorectal adenomatous polyposis can be caused by other hereditary diseases, including polymerase proofreading-associated polyposis [15], *NTLH1* tumor syndrome [16], and *MBD4*-associated neoplasia syndrome [17]. Pathogenic biallelic germline variants in one of the four mismatch repair genes (*MLH1, MSH2, MSH6,* or *PMS2*) can cause colorectal adenomatous polyposis in childhood [18]. In our case, we performed germline multigene panel testing to detect genes associated with these diseases with blood samples; however, no pathogenic variants suggestive of these diseases were found in the germline. Pathogenic biallelic germline variants in *MSH3* are also associated with colorectal polyposis, namely, *MSH3*-associated polyposis [19]. In the present case, we identified a heterozygous variant in *MSH3*. Although it was expected to be silent, we tried to annotate the variant; in the tissue immunostaining, the expression of MSH3 was the same in the normal mucosa, adenomas, and carcinomas, suggesting that the *MSH3* variant in our case was unlikely to have contributed to the development of polyposis or carcinogenesis. Germline loss-of-function variants in *AXIN2* are also reported to be associated with colonic polyposis and colorectal cancers [20]. We added the mRNA sequence of *AXIN2* using blood samples, and the function of *AXIN2* was deemed to be normal. In addition to germline variants related to adenomatous polyposis, loss-of-function of *RNF43* can cause serrated polyposis syndrome [21]. In the present case, serrated changes were observed in part of the polyps. However, no obvious variants in the *RNF43* gene sequence were detected. Overall, no pathogenic variants were detected in the present case, and we diagnosed the patient with CPUE. The sporadicity and localization of the lesions indicated the possibility of the involvement of somatic variants, such as *APC* mosaicism [22]. In our case, the variant frequency threshold was lowered to 0.5% for *APC* to investigate the possibility of mosaic, but no variants were detected as possible causes from blood samples. Mosaicism might be detected by genetic searches in adenomas and surrounding normal mucosa. In addition to genetic factors, colorectal polyposis has been associated with cancer treatment in childhood or adulthood [23, 24]; however, no such history was found in our patient.

Herein, we reported the first CPUE case with numerous adenomas and multiple adenocarcinomas, all located in the right side of the colon. The diagnosis of CPUE was made after a germline multigene panel testing was conducted on a blood sample to detect polyposis-associated germline variants. However, no pathogenic variant was detected. The sporadicity of the case and localization of the lesions raised the possibility of the involvement of somatic variants. Given that CPUE cases may encompass some undiagnosed hereditary diseases, it is generally accepted that the standard treatment for CPUE cases with numerous adenomas is total proctocolectomy. However, in this specific case, considering the patient’s general condition and distribution of the lesions, we opted to perform right hemicolectomy and regional lymph node dissection as a curative treatment. This approach resulted in a long-term recurrence-free survival for the patient. Therefore, in cases with localized lesions, segmental colectomy might be an option for curative treatment.

## Conclusions

We present a rare case of CPUE involving numerous adenomatous polyposis and multiple carcinomas localized in the right side of the colon. The patient’s condition was successfully treated with right hemicolectomy and regional lymph node dissection, and long-term recurrence-free survival was achieved. Genetic analysis using blood samples did not reveal a causative germline variant related to polyposis. Segmental colectomy might be a curative approach in CPUE cases with localized lesions.

## Data Availability

The study data can be made available upon any reasonable request.

## Abbreviations

FAP: Familial adenomatous polyposis
CPUE: Colonic adenomatous polyposis of unknown etiology
